# Evaluation of growth adaptation of *Cinnamomum camphora* seedlings in ionic rare earth tailings environment

**DOI:** 10.1038/s41598-023-44145-z

**Published:** 2023-10-07

**Authors:** H. Zhang, C. Liu, X. Lu, G. Xia

**Affiliations:** 1 Jiangxi Provincial Engineering Research Center of Seed-Breeding and Utilization of Camphor Trees, Nanchang Institute of Technology, Nanchang, China; 2 Yao Hu Honor School Nanchang Institute of Technology, Nanchang, China; 3 Jiangxi Provincial Technology Innovation Center for Ecological Water Engineering in Poyang Lake Basin, Nanchang, China

**Keywords:** Ecology, Ecology, Environmental sciences

## Abstract

The root system is an important organ for nutrient uptake and biomass accumulation in plants, while biomass allocation directly affects essential oils content, which plays an essential role in plant growth and development and resistance to adverse environmental conditions. This study was undertaken to investigate the differences and correlation of biomass allocation, root traits and essential oil content (EOC), as well as the adaptations of camphor tree with different chemical types to the ionic rare earth tailing sand habitats. Data from 1-year old cutting seedlings of *C. camphora* showed that the biomass of *C. camphora* cuttings was mainly distributed in root system, with the ratio of root biomass 49.9–72.13% and the ratio of root to canopy 1.00–2.64. The total biomass was significantly positively correlated with root length (RL), root surface area (RSA) and dry weight of fine roots (diameter ≤ 2 mm) (*P* < 0.05). Root biomass and leaf biomass were negatively and positively with specific root length (SRL) and specific root surface area (SRSA), respectively. Leaf biomass presented a positive effect on EOC (*P* < 0.05), with the correlation coefficient of 0.808. The suitability sort of these camphor trees was as follows: *C. camphora* β-linalool, *C. camphora* α-linaloolII, *C. camphora* α-linaloolI being better adapted to the ionic rare earth tailings substrate, *C. camphora* citral being the next, and *C. porrectum* β-linalool and *C. camphora* borneol being the least adaptive. EOC played a positive role in the adaptation of *C. camphora* (*R*^2^ = 0.6099, *P* < 0.05). Therefore camphor tree with linalool type is the appropriate choice in the ecological restoration of ionic rare earth tailings. The study could provide scientific recommendations for the ecological restoration of ionic rare earth tailings area combined with industrial development.

## Introduction

Ionic rare earth ores, resulting from granite weathering in subtropical climate conditions, are an important strategic resource and mainly distributed in south China. However, the scarcity of ionic rare earth ore resources and the high pollution of the mining process caused a great waste of ionic rare earth resources and increasingly serious ecological and environmental problems^1^. Mine vegetation restoration measures to improve the ecological environment of ionic rare earth tailings is an urgent problem to be solved. At present, most of the plants used for the remediation of ionic rare earth tailings area have low economic value, shallow root systems and weak soil-fixing capacity. These plants have low water and fertilizer use efficiency, resulting in surface source pollution caused by the loss of large quantities of fertilizer with soil and water. Therefore, suitable plants with a developed root system, strong stress resistance, and high economic value can better meet the needs of vegetation restoration in mining areas. In previous studies on the selection of suitable plants for ionic rare earth tailings, the authors found that under high temperature and drought conditions, camphor tree seedlings have higher water use efficiency and adaptability around the ionic rare earth tailings compared to the native plants *Liquidambar formosana* and *Schima superba*^2^, and could be used for vegetation restoration in ionic rare earth tailings area.

Cinnamomum spp. and Litsea spp., an important local economic plant^3,4^, contains a variety of essential oils^5^. According to the main components of the essential oils, camphor trees are divided into different chemical types, such as *C. camphora* α-linalool, *C. camphora* β-linalool, *C. camphora* borneol, *C. camphora* brain, *C. camphora* eucalyptus, *C. camphora* citral, etc. Among them, the camphor tree whose main component of essential oil is not clear is called *C. camphor* miscellaneous. According to previous researches, the same camphor tree could have different chemical types, such as camphor type, borneol type, 1,8-cineole type, α-rolinalool type, citral type, isonerolidol type, safrole type, and other chemotypes^6^. In addition, different camphor tree species may also have the same chemotype. For example, *Cinnamomum camphora*, *Cinnamomum bodinieri*, *Cinnamomum porrectum*, and *Cinnamomum tenuipilum* all have citral chemotypes^7^. At present, *C. camphora* are grown under dwarf cultivation in large areas in the southern provinces in China, and the aboveground parts of the stubble plants are extracted every year to extract essential oils. Therefore, the biomass allocation characteristics of *C. camphora* directly affect the production of essential oils.

Root system is an important organ for plants to absorb nutrients, playing a vital role in the biomass accumulation. Fine root (diameter ≤ 2 mm) traits (root length (RL), root surface area (RSA), specific root length (SRL), etc.) have proven to be important indicators of root morphology and structure, ecological adaptability and resource acquisition capacity. These indicators play a crucial role in nutrient uptake and transportation^8–10^. It has been shown that root architecture is strongly influenced by the environment and adapts to changing environments through root plasticity^11,12^. Plants with high carbon investments in poor soils allocate more biomass to fine roots for root synthesis and access to nutrients by increasing RL^13,14^, while plants with low carbon investments uptake through morphological and physiological plasticity of the root system^15^, implying a trade-off between resource foraging and resource conservation^14^ In contrast, under eutrophic conditions, plants absorb soil nutrients by increasing fine root SRL and RSA and decreasing RL^16,17^. Some studies also showed that root dry weight, fine root RL and fine root RSA were all significantly and positively correlated with yield in soybean at bulging stage under water-nitrogen treatment^18^. Fast-growing species have higher SRL and lower root tissue density than slow-growing species^19^. These inconsistent results may be related to soil infertility and plant species.

The chemical composition and structure of the soil strongly affect the growth and development of plants. Ecophysiologically, the effects of soil are translated in changes in plant primary and secondary metabolism (essential oils)^20^. Therefore it is necessary to screen the chemotypic camphor species with high adaptability and high essential oil content (EOC) for the ionic rare earth mine tailings area. On this basis, we provide the following assumptions: (1) there are diversity in the root morphological characteristics of camphor tree seedlings with different chemotypic essential oils in ionic rare earth backfilling matrix; (2) root morphological characteristics (such as RL, RSA, SRL, etc.) play a vital role in the biomass allocation of camphor tree seedlings; (3) the essensial oils in camphor tree seedlings help enhance the adaptability of plants to ionic rare earth backfilling. In order to solve the above assumption, we compared and analyzed the biomass allocation, root traits and EOC of 7 different chemical types of camphor tree seedlings in the ionic rare earth tailings matrix, and illustrated the relationship between root morphology and biomass allocation of camphor tree seedlings, and comprehensively analyzed the ecological and economic adaptations of different chemical camphor trees. The results of the study could provide scientific recommendations and management strategies for the ecological restoration of ionic rare earth tailings combined with industrial development.

## Results

### Plant growth status

The plant height of No. 5 and No. 6 camphor trees was significantly higher than that of No. 3 and No. 7 camphor trees, while there was no significant difference with miscellaneous camphor trees. Camphor No. 1, No. 5 and No. 6 had 32.5–60.3% higher crowns than the other chemotypes (Table 1).

**Table 1:** Growth status of camphor seedlings.

No	Chemical type	Height (cm)	Average crown (cm)
1	*C. camphora* miscellaneous	34.67 ± 3.60ab	33.35 ± 5.60a
2	*C. camphora* citral	29.86 ± 4.25bc	20.81 ± 4.94b
3	*C. camphora* borneol	28.65 ± 4.27c	21.30 ± 5.77b
4	*C. camphora* α-linalool I	30.62 ± 4.47abc	22.94 ± 5.22b
5	*C. camphora* α-linalool II	35.56 ± 4.94a	31.43 ± 6.76a
6	*C. camphora* β-linalool	35.76 ± 7.72a	30.39 ± 7.53a
7	*C. porrectum* β-linalool	21.19 ± 6.98d	21.89 ± 7.91b

Each value represents the mean ± SE. Means within a column followed by the same letter(s) are not significantly different (*P* > 0.05).

### Biomass allocation

The leaf biomass in No. 5–7 *C. camphora* was significantly higher than that of No. 1–3 camphor trees. The No. 1 *C. camphor* miscellaneous had the most stem, root and total biomass, followed by No. 4–6 camphor trees and No. 7 *C. porrectum* β-linalool was the lowest, in which the highest was 5.44, 3.96 and 3.91 times the lowest (Table 2).

**Table 2:** Biomass allocation characteristics of camphor seedlings with different chemical types.

No	Chemical type	Leaf	Stem	Root	Biomass (g)	Root-shoot ratio
		Biomass (g)	Biomass (g)	Biomass (g)		
1	*C. camphora* miscellaneous	0.55 ± 0.08b	6.26 ± 0.94a	18.5 ± 2.43a	25.31 ± 3.09a	2.64 ± 0.53a
2	*C. camphora* citral	0.59 ± 0.12b	2.22 ± 0.94cde	8.00 ± 0.85bc	10.81 ± 2.40b	2.12 ± 0.69ab
3	*C. camphora* borneol	0.60 ± 0.14b	2.08 ± 0.83de	6.00 ± 0.28 cd	8.68 ± 1.55b	2.43 ± 0.55a
4	*C. camphora* α-linalool I	0.61 ± 0.14b	3.48 ± 1.50bcd	7.57 ± 0.15bcd	11.66 ± 0.39b	1.31 ± 0.12c
5	*C. camphora* α-linalool II	0.66 ± 0.16ab	3.63 ± 1.71b	7.63 ± 1.25bcd	11.92 ± 3.01b	1.25 ± 0.37c
6	*C. camphora* β-linalool	0.75 ± 0.20a	2.83 ± 1.13bc	9.25 ± 1.20b	12.83 ± 2.08b	1.00 ± 0.04c
7	*C. porrectum* β-linalool	0.66 ± 0.22ab	1.15 ± 0.74e	4.67 ± 1.59d	6.48 ± 0.27c	1.95 ± 0.19b

Each value represents the mean ± SE. Means within a column followed by the same letter(s) are not significantly different (*P* > 0.05).

**Figure 1:**
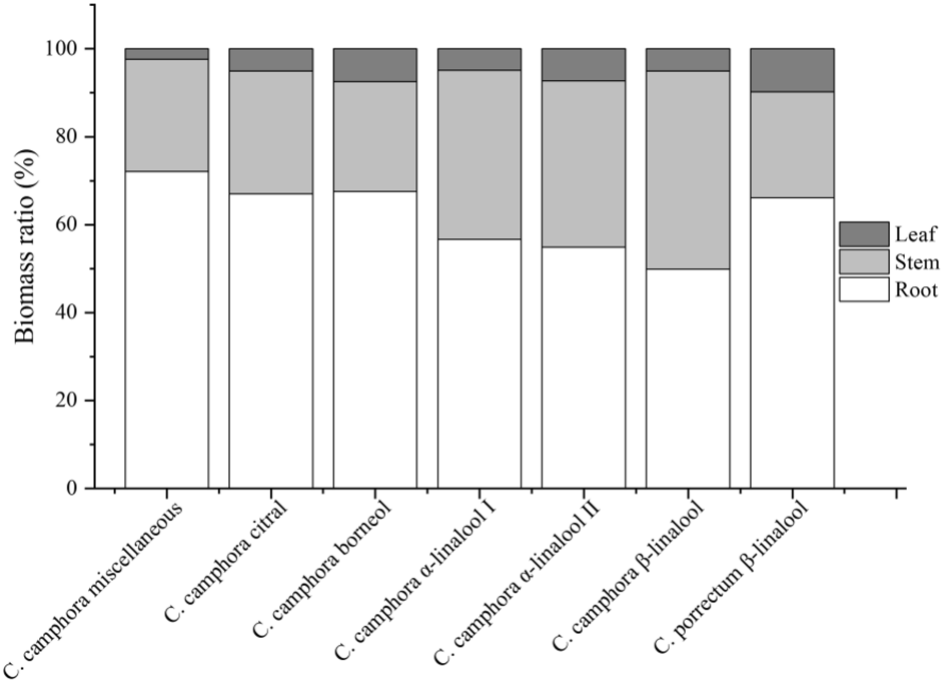
Biomass allocation ratio of different organs in camphor seedlings with different chemical types.

In terms of biomass distribution, the biomass of all camphor trees was mainly distributed in the root system, with a distribution ratio of 49.9–72.13%. The biomass in leaf accounted for 2.41–9.81% and that in stem accounted for 24.09–45.08%. Among them, the highest proportions of root biomass and leaf biomass were No. 1 camphor, and No. 7 *C. porrectum* β-linalool, respectively (Fig. 1). The root-to-shoot ratios of the 7 species of camphor trees ranged from 1 to 2.64, with that in No. 1 *C. camphor* miscellaneous was the highest (Table 2).

### Root traits

#### RL and RSA

Compared with No. 1 *C. camphor* miscellaneous, the RL and RSA of fine and coarse roots of other chemotypes of camphor trees were significantly reduced, in which the RL of fine and coarse roots was reduced by 20.9–83.6%, respectively, and RSA reduced by 26.9–83.3%, respectively. The RSA of fine roots was significantly higher in chemotype camphor No. 3 and No. 6 than in No. 4, No. 5 and No. 7, and lowest in No. 2 camphor. In terms of root length ratio, The ratios of the seven chemotypes ranged from 50.82 to 482.49, with the highest root length ratio in No. 3 *C. camphor* borneol (Table 3).

**Table 3:** Root length and root surface area of camphor seedlings of different chemical types.

No	Chemical type	RL (mm)		Ratio of RL	RSA (cm^2^)		Ratio of RSA
		Fine root	Coarse root		Fine root	Coarse root	
1	*C. camphora* miscellaneous	7844.65 ± 449.09a	45.23 ± 9.26a	157.68 ± 0.08c	1040.56 ± 59.83a	68.49 ± 1.41a	16.04 ± 1.76bc
2	*C. camphora* citral	1283.70 ± 115.94d	9.53 ± 2.13bc	50.82 ± 8.93d	235.69 ± 22.31d	8.16 ± 1.93d	22.24 ± 1.21a
3	*C. camphora* borneol	4368.28 ± 351.13c	8.3 ± 2.10c	482.49 ± 16.23a	556.39 ± 49.02c	26.28 ± 12.64bc	17.33 ± 0.77b
4	*C. camphora* α-linalool I	1769.09 ± 140.78d	25.85 ± 11.36bc	78.57 ± 24.8d	243.23 ± 23.51d	46.89 ± 16.01b	4.83 ± 0.80d
5	*C. camphora* α-linalool II	6205.74 ± 69.94b	25.18 ± 8.72bc	273.15 ± 35.90b	760.21 ± 63.52b	38.79 ± 4.18 cd	22.02 ± 2.79a
6	*C. camphora* β-linalool	5813.33 ± 28.96b	27.86 ± 10.32ab	176.84 ± 15.45c	610.90 ± 63.05c	45.36 ± 6.64b	13.55 ± 0.32c
7	*C. porrectum* β-linalool	1373.67 ± 312.33d	21.71 ± 1.04bc	70.23 ± 14.54d	174.06 ± 20.59d	29.61 ± 3.88bcd	5.93 ± 1.17d

Each value represents the mean ± SE. Means within a column followed by the same letter(s) are not significantly different (*P* > 0.05). *RL* root length, *RSA* root surface area, ratio of RL was the ratio of fine root length to coarse root length, *ratio of RSA* the ratio of fine root surface area to coarse root surface area.

#### Root weight

The dry weight of fine roots and coarse roots of the No. 1 *C. camphor* miscellaneous was significantly higher than that of other camphor trees, and the weight of fine roots was about 3–5 times that of other camphor trees. There was no significant difference between the weights of fine roots of other camphor trees. In terms of fine-coarse root weight ratio, that of No. 1, 3, 5, 6, and 7 camphor trees ranged from 0.91 to 1.53, with no significant differences (Table 4).

**Table 4:** Biomass of Fine and coarse roots in camphor trees with different chemical types.

No	Chemical type	Root biomass (g)		Fine-coarse root weight ratio
		Fine root	Coarse root	
1	*C. camphora* miscellaneous	11.20 ± 0.27a	7.30 ± 1.30a	1.53 ± 0.29a
2	*C. camphora* citral	2.95 ± 0.78b	5.05 ± 0.26b	0.58 ± 0.15b
3	*C. camphora* borneol	3.30 ± 0.42b	2.70 ± 0.14c	1.22 ± 0.22a
4	*C. camphora* α-linalool I	2.25 ± 0.21b	5.32 ± 0.07b	0.42 ± 0.05b
5	*C. camphora* α-linalool II	3.90 ± 0.70b	3.73 ± 0.60bc	1.05 ± 0.10a
6	*C. camphora* β-linalool	4.40 ± 0.14b	4.85 ± 1.06b	0.91 ± 0.17a
7	*C. porrectum* β-linalool	2.47 ± 0.81b	2.20 ± 0.92c	1.12 ± 0.08a

Each value represents the mean ± SE. Means within a column followed by the same letter(s) are not significantly different (*P* > 0.05).

#### SRL and SRSA

The SRL and SRSA reflect the ability of plants to absorb nutrient resources. The SRL of No. 2, No. 3, No. 5, and No. 6 camphor trees was significantly higher than that of No. 1, No. 4 and No. 7 camphor trees, with the ratio 36.8–110.9%. Among them, the fine root of the No. 5 camphor tree had the largest SRL. For the ratio of SRL and SRSA varied in the range of 87.88–400.6 and 43.56–105.15, respectively. The SRSA of the fine roots and the whole roots of No. 3 and No. 5 camphor trees were significantly higher than those of other camphor trees (Table 5).

**Table 5:** Specific root length and specific surface area of camphor seedlings of different chemical types.

No	Chemical type	SRL (cm/g)		Ratio of SRL	SRSA (cm^2^/g)		
		Fine root	Coarse root		Fine root	Coarse root	Total root
1	*C. camphora* miscellaneous	96.59 ± 5.18c	0.45 ± 0.07cd	163.41 ± 0.26bc	109.40 ± 12.19cd	8.37 ± 1.46b	63.29 ± 2.44bc
2	*C. camphora* citral	169.61 ± 6.04a	0.43 ± 0.03cd	125.98 ± 60.42c	87.75 ± 35.25de	1.93 ± 0.60c	43.56 ± 8.63d
3	*C. camphora* borneol	132.78 ± 6.43ab	1.08 ± 0.09a	400.60 ± 85.46a	170.97 ± 36.84ab	9.62 ± 4.18ab	97.46 ± 14.87a
4	*C. camphora* α-linalool I	82.60 ± 8.02c	0.30 ± 0.05d	100.75 ± 24.52c	111.99 ± 16.62cd	10.64 ± 1.83ab	38.77 ± 2.81d
5	*C. camphora* α-linalool II	174.18 ± 39.03a	0.63 ± 0.12bc	252.80 ± 7.12b	197.49 ± 21.57a	9.36 ± 0.38ab	105.15 ± 8.82a
6	*C. camphora* β-linalool	132.18 ± 3.59ab	0.57 ± 0.06bc	237.54 ± 43.93b	140.12 ± 11.90bc	9.43 ± 0.69ab	71.70 ± 0.80b
7	*C. porrectum* β-linalool	93.11 ± 9.25c	0.86 ± 0.14ab	87.88 ± 1.24c	54.38 ± 9.85e	14.46 ± 5.24a	53.73 ± 8.66cd

Each value represents the mean ± SE. Means within a column followed by the same letter(s) are not significantly different (*P* > 0.05). *SRL* specific root length, *SRSA* specific root surface area, *Ratio of SRL* the ratio of SRL of fine root to coarse root.

### EOC

With the exception of *C. camphora* miscellaneous, the oil yield of the leaves of the six different chemotypes of camphor was 0.42–2.88%, and the EOC was 2.25–3.02 mg/plant. The highest EOC was found in chemotypes 5 and 6, which were 8.2%, 11.6% and 30.2%, 34.2% higher than chemotypes 2 and 3, respectively. All the above shows that the EOC of linalool-type camphor is relatively higher in the ionic rare earth tailing sand matrix (Table 6).

**Table 6:** Essential oil content of camphor trees with different chemical types.

No	Chemical type	EOC(mg/plant)	Oil yield (%)	Essential oil composition
1	*C. camphora* miscellaneous	2.05 ± 0.12b	0.40c	–
2	*C. camphora* citral	2.32 ± 0.08b	0.79c	Citral
3	*C. camphora* borneol	2.25 ± 0.06b	0.42c	Borneol
4	*C. camphora* α-linalool I	2.41 ± 0.11b	2.41ab	α-Linalool
5	*C. camphora* α-linalool II	2.51 ± 0.07ab	2.73a	α-Linalool
6	*C. camphora* β-linalool	3.02 ± 0.13a	2.88a	β-Linalool
7	*C. porrectum* β-linalool	2.39 ± 0.08b	1.73b	β-Linalool

Each value represents the mean ± SE. Means within a column followed by the same letter(s) are not significantly different (*P* > 0.05). *EOC* essential oil content.

### Relationship between plant biomass, EOC and root traits indexes

**Figure 2:**
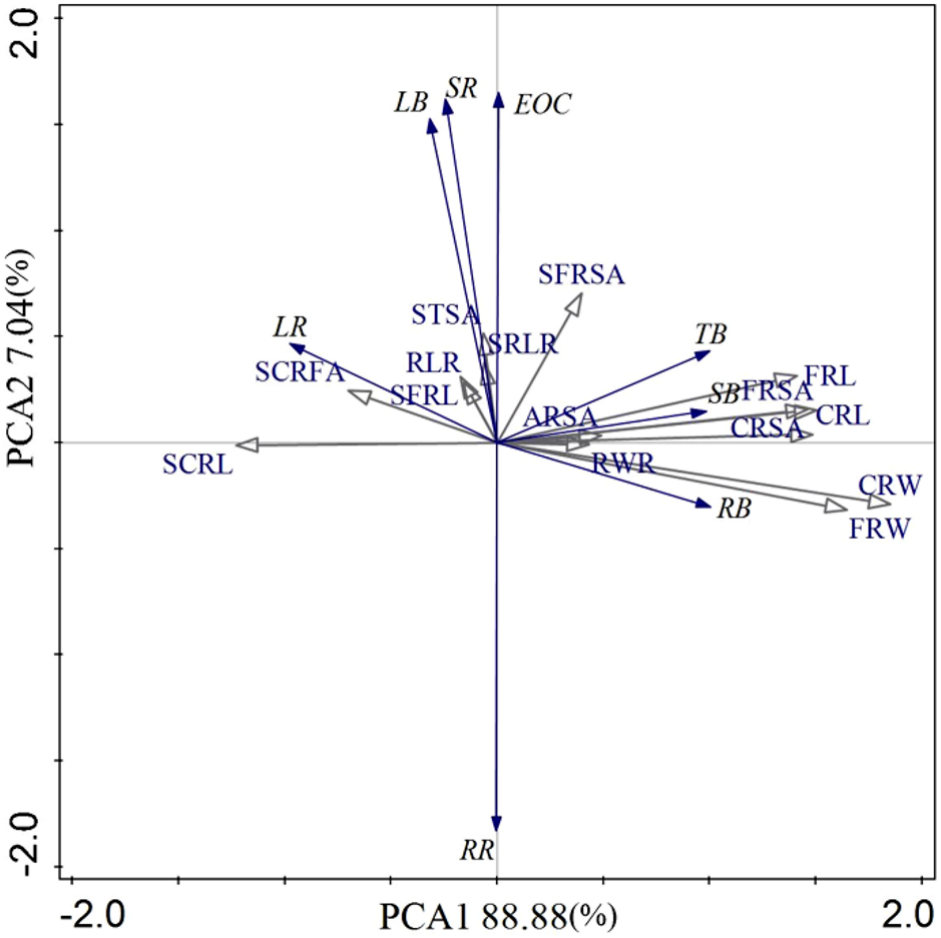
PCA (principal component analysis) among the plant biomass, root traits indexes and essential oil content in camphor seedlings of different chemical types. *TB* total biomass, *SB* stem biomass, *SR* stem ratio, *LB* leaf biomass, *LR* leaf ratio, *RB* root biomass, *RR* root ratio, *EOC* essential oil content, *FRL* fine root length, *CRL* coarse root length, *RLR* root length ratio of fine root to coarse root, *FRSA* fine root surface area, *CRSA* coarse root surface area, *SRSA* specific root surface area, *FRW* fine root weight, *CRW* coarse root weight, *RWR* root weight ratio of fine root to coarse root, *SFRL* specific fine root length, *SCRL* specific coarse root length, *SRLR* specific root length ratio of fine root to coarse root, *SFRSA* specific fine root surface area, *SCRSA* specific coarse root surface area, *STRAS* specific total root surface area. The blue arrows represent plant biomass and essential oil content, the gray arrows represent root traits.

There was a significant and positive correlation between the total biomass (TB) and several root characteristics, including RL, RSA, and root biomass (RB) (*P* < 0.05) as well as stem biomass (SB) (*P* < 0.01). Both RSL and SRSA exhibited a negatively effect on RB and a positive effect on leaf biomass (LB) (Fig. 2). The results show that camphor mainly accomplishes biomass accumulation by establishing root system, extending RL and expanding RSA, rather than changing root system diameter to achieve this purpose. Through the above process, camphor tree completed the adaptation to the ionic rare earth tailings environment.

Principal Component Analysis was conducted to evaluate the relationship between plant biomass, EOC and root traits index (Fig. 2). PCA1 accounted for 88.88% of the variations, while PCA2 accounted for 7.04%. Of the evaluated attributes, the coarse root weight (CRW) had significant conditional effects (*P* < 0.05), while the fine root surface area (FRSA) had extremely significant effects (*P* < 0.01). In addition, the EOC was positively correlated with LB and SR. The multiple linear regression results showed that EOC = − 0.247 + 3.652LB + 1.057SR (*F* = 68.573, *P* < 0.01). The results of PCA showed that the suitability sort of camphor trees with main chemical types were in the order of *C. camphora* β-linalool > *C. camphora* α-linalool II > *C. camphora* α-linalool I > *C. camphora* citral > *C. porrectum* β-linalool > *C. camphora* borneol.

### Relationship between adaptive ranking and EOC

**Figure 3:**
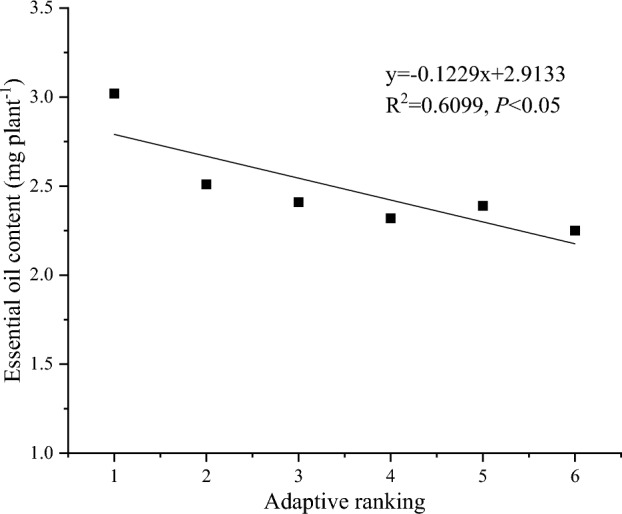
Relationship between adaptive ranking and essential oil content (EOC) of different chemotypes camphor in ionic rare earth tailing sand substrates.

Different chemotypes of camphor were ranked in order of their adaptability to ionic rare earth tailings from high to low (1–6), and the serial numbers were plotted against the essential oil content as shown in Fig. 3. It showed that as the adaptability decreased, the EOC decreased, and the two indicators were significantly and linearly correlated with each other (*P* < 0.05) (Supplementary Information).

## Discussion

Biomass accumulation indicates the ability of plants to utilize and accumulate space resources, and its distribution characteristics can reflect adaptation strategies of plant to environment^21–23^. In this study, the soil structure of the ionic rare earth tailings was damaged, and the water retention capacity was poor, as well as the nutrients were poor^24^. Thus, plants were under severe water and nutrient stress. All camphor trees had the highest biomass in the root, followed by stems, and the leaf had the lowest biomass (Table 2, Fig. 1), indicating that camphor tree seedlings allocated more resources to the root system to obtain nutrients in order to cope with the unfavorable conditions of ionic rare earth tailings. Among the tested plants, the roots and total biomass of *C. camphor* Miscellaneous were significantly higher than those of other chemotype camphor trees (Table 2), indicating that the *C. camphor* Miscellaneous had better adaptability and growth potential to the ionic rare earth tailings matrix by increasing root length, root surface area and root biomass, and dedicating more resources to root system construction to improving adaptation to the ionic rare earth tailings environments. It is mainly because these essential oils are secondary metabolites of camphor tree, and the synthetic precursors from carbohydrates produced by photosynthesis, mainly affects the synthesis and accumulation of secondary metabolites by affecting the synthesis and accumulation of primary metabolites^25^. Therefore, the accumulation of secondary metabolites in camphor tree with typical chemical substances consumes some photosynthetic products, and the substances used for plant biomass accumulation and distribution would be limited^26–28^. For this reason, the biomass accumulation of all chemical camphor trees was lower than that of *C. camphor* Miscellaneous.

It was shown that the root proportion and root-to-shoot ratio of *C. camphora* citral and *C. porrectum* β-linalool were significantly higher than those of *C. camphora* α-linalool and *C. camphora* β-linalool (Fig. 2), indicating that in the ionic rare earth tailings matrix, the growth of *C. camphora* citral and *C. porrectum* β-linalool may be severely stressed. As a result, the plants used more resources for building root systems and improved the adaptability of plants to ionic rare earth tailings by increasing the root-to-shoot ratio^29–31^, which was consistent with Wang et al.^32^ on the anatomical structure characteristics and drought resistance characteristics of leaves of camphor trees for different chemical types of oil. The drought resistance of *C. camphora* citral and *C. porrectum* β-linalool was lower than that of linalool (α-linalool and β-linalool type), and they were vulnerable to drought stress under the condition of low water retention in ionic rare earth tailings. The proportion of leaf biomass in *C. porrectum* β-linalool was significantly higher than in other camphor trees (Fig. 2), mainly because the leaf area of sassafras (*C. platyphyllum*) was larger than that of other chemotype camphor trees, which required more resources for leaf growth and photosynthesis and promoted the accumulation of leaf biomass, which was another reason for weaker adaptivity in *C. porrectum* β-linalool than other chemical type camphor.

Most studies have shown that the more biomass allocated to fine roots indicates that the root system can better absorb nutrients, potentially increasing the ability of plants to adapt to the soil environment^33–35^. In the current study, the large differences in the biomass of different tissues and their percentage, as well as in the root indexes among different chemotypes of camphor (Tables 2, 3, 4, 5) indicated that there was great variance in the adaptability of these camphor trees to the ionic rare earth tailing sand substrate due to the factors of plant species and chemotypes. The PCA results showed that total biomass was significantly and positively correlated with RL, RSA and dry weight, while root biomass and leaf biomass were negatively and positively correlated with fine root SRL and SRSA, respectively. The RL, RSA, root weight and SRSA of the fine roots of *C. camphora* citral, *C. camphora* α-linaloolIand *C. porrectum* β-linalool are the lowest, which limited the use of carbon sources in the soil^36^, In comparison with *C. camphora* α-linalool I and *C. porrectum* β-linalool, *C. camphora* citral with higher SRL of fine root had a higher capacity to absorb nutrients in soil^31^. However, *C. camphora* citralr transported absorbed nutrients to the leaves for photosynthesis, and root system construction is compromised, making it the least adapted to ionic rare earth tailings.

The composition of essential oils with different chemotypes is an expression of biodiversity within plant species^37^. As the secondary metabolites, essential oils play an important role in plant growth and development and resisting external adverse environment^38^. Previous studies have shown that the secondary metabolite essential oil produced by camphor belongs to terpenes, and the biosynthetic pathway of terpenes is usually divided into three stages: the formation stage of C5 precursor isopentenyl diphosphate (IPP) and its double bond isomer dimethylallyl diphosphate (DMAPP); The generation stage of direct precursors (farnesyl diphosphate FPP, tauryl diphosphate GPP, tauryl tauryl diphosphate GGPP, etc.)^39^; Terpene formation and modification stage (redox, acylation, glycosylation, etc.). Among the three stages, the first two stages are shared by all terpenoids, and the third stage determines the structural diversity of terpenoids^40^. It could be seen that differences in the formation pathways of essential oils from different chemotypes of camphor^41^. In addition, the adaptability of *C. camphora* citral to ionic rare earth tailings was lower than that with linalool type and borneol type, which might be due to the difference in metabolic pathways and energy consumption of secondary metabolites, such as citral, borneol and linalool produced by camphor trees, so that camphor trees with different chemical type had differences in tissue construction and biomass distribution. However, the pathway of natural secondary metabolites in camphor tissue is not clear, which needs to be further revealed.

*C. camphora* in this study were enriched with different types and contents of essential oils (Table 6), and their adaptability to the ionic rare earth tailings substrate increased with increasing essential oil content (Fig. 3). Lajayer et al. (2017) ***made similar observations that low levels of copper and zinc stress promoted carbon sources utilised in the terpene biosynthetic pathway, may positively influence essential oil quality and quantity^42–44^. In some cases, stressed plants are induced to change the quantity and quality of their secondary metabolites, such as phenolic compounds and essential oils, in order to cope and adapt to the concomitant environment. All of the above suggests that the amount of essential oil content may be one of the strategies used by Camphor to cope with ionic rare earth tailings habitats.

In summary, the adaptation of different chemotypes of camphor to ionic rare earth tailings sand habitats is a complex process that requires biomass partitioning between above- and below-ground tissues and among different organs, and even EOC. Our experimental results confirmed the pre-hypothesis. The conclusions were as follows. Camphor accumulates total biomass by increasing RL, RSA and dry weight, and increases the capacity of fine roots to absorb nutrients by altering root diameter indexes (SRL, SRSA), which is used for leaf biomass accumulation aboveground. Preliminary evaluation of the adaptability of different chemotypes of camphor seedlings in ionic rare earth tailings sand resulted in *C. camphora* β-linalool, *C. camphora* α-linalool II and *C. camphora* α-linalool I being better adapted to the ionic rare earth tailings sand substrate, *C. camphora* citral being the next best, and *C. porrectum* β-linalool and *C. camphora* borneol being the least well adapted. Plant adaptation is positively correlated with plant EOC, i.e., an increase in the EOC of camphor had a positive effect on its adaptation to ionic rare earth tailings. Therefore, *C. camphora* with linalool type could be considered as economic and industrial trees in the vegetation restoration in combined with industrial development in the ionic rare earth tailings area.

## Materials and methods

### Experimental materials

The tested plants were 1-year-old *Cinnamomum camphora* cuttings of excellent chemotypic monocots pre-screened by the subject group of Jiangxi Province Engineering Research center of Seed-breeding and Utilization of Camphor Trees. The group collected wild camphor seeds from the southern provinces of China (wild distribution area of *Cinnamomum camphora*) in 2018. In the second year, the seeds were sown to obtain live seedlings of different seed sources, after which the leaves of the live seedlings were collected. Then the phytochemical type was determined by the odor method, and the essential oil was extracted. The relative content of the main components in the leaf essential oil was detected by the water distillation method and the gas chromatography–mass spectrometry method. Finally, the 3-year old live seedlings of Camphoria with a high EOC and high content of the main components were taken as cuttings with the 1-year old semi-lignified branches. The cuttings were finally obtained and used as test materials for this experiment. The tested plants were sourced in compliance with relevant institutional, national and international guidelines and legislation.

The tested soil was obtained from an abandoned ionic rare earth mine in Xunwu County, Jiangxi Province in China. The physical and chemical data of the tested ionic rare earth tailings were as follows: soil pH was 5.08 (strongly acidic); the conductivity was as high as 79.12 dS/m; the soil organic carbon, organic matter, total nitrogen, and total phosphorus contents in the tailings were 6.29 g/kg, 10.85 g/kg, 0.20 mg/kg and 1.21 g/kg, respectively; and the available phosphorus and available potassium contents were 13.78 mg/kg and 225.97 mg/kg, respectively.

### Experiment method

The experiment was carried out in the Biotechnology Test Base of Nanchang Institute of Technology in Nanchang, Jiangxi Province in China. The base belongs to the subtropical humid monsoon climate, with annual rainfall of 1600–1700 mm, rainy season from April to June, seasonal drought period from July to September, annual average relative humidity of 78.5%, and annual sunshine duration of 1723–1820 h.

Based on the excellent camphor tree species screened by the research group based on "three highs" (high biomass, high essential oil content, and high content of main components), 1-year-old cutting seedlings of camphor trees for each chemical type (about 15 cm in height, 12 cm in average crown width, and about 5.5 mm in basal diameter) were selected and planted in pots (29 cm in diameter, 26 cm in height) in March 2020. Each pot was filled with 8 kg of ionic rare earth tailings matrix, and there were 10 pots of camphor trees for each chemical type, totaling 70 pots. The initial stage of the experiment was in the local rainy season, and the potted plants were grown under natural conditions. During the high temperature and dry period in July and August, the water was completely replenished every 10 days, and the water was sufficient when it flowed out from the bottom of the pots.

#### Biomass determination

At the end of the plant growing season, all the plants for each chemotype were harvested, and the plant height and crown width were measured. We removed the aerial parts of the plants uniformly, and then divided each camphor tree into stems and leaves and put them into an envelope and marked them, respectively. The samples were baked at 80 °C to constant weight to obtain the leaf and stem biomass of the plant. The sum of the leaf and stem biomass is called the above-ground biomass.

#### Root trait determination

The whole plant was excavated using the "whole root method". Along the direction of each root, we used tweezers and a small shovel to get the whole root system of the plant, rinsed it with clean water in a mesh bag, and divided the root system into fine roots (diameter ≤ 2 mm) and coarse roots (diameter > 2 mm). Through the root analysis system WinRHIZO Pro software (Reagent Instruments, Quebec, Canada), the RL and RSA of the plants were obtained. Afterward, the roots were scanned, put into envelopes, and baked at 80 °C to constant weight. The ratio of root length to root weight is called SRL, and the ratio of RSA to root weight is called SRSA. The sum of biomass of fine and coarse roots is called the underground biomass.

#### Essential oil content determination

All leaves of camphor tree were collected and grouped in November according to chemical type, and then took about 200 g fresh samples of the leaves from different chemical types. The essential oil was obtained by steam distillation through the patented product "Portable camphor essential oil steam distiller" developed by Jiangxi Province Camphor Breeding and Development and Utilization Engineering Research Center of Nanchang Engineering College. At the same time, the water content of leaves was measured quickly by a rapid moisture meter (Sartorius MA150). The amount of essential oil/dry mass of leaf biomass was used as the index of essential oil content and the essential oil yield of the total yield per plant were also calculated based on the dry mass per plant^29,45,46^.

### Statistical analysis

One-way analysis of variance (one-way ANOVA) was used to compare the significance of differences in growth status, biomass distribution, and root traits of camphor tree seedlings with different chemotypes. The Duncan method was used for multiple comparisons, and the significance level was set at *P* < 0.05. All data were reported as mean ± SE from 10 plants per chemical type of camphor tree. Correlations between the plant biomass and the root trait indexes, and that of the essential oil content with the biomass and root morphology indexes were obtained by Pearson's analysis, and the analysis results were shown in the form of an annex. The principal component analysis (PCA) was used to comprehensively evaluate the relationship between biomass allocation indexes, root traits parameters and EOC of plants, as well as the adaptive of plants to the ionic rare earth tailings. The multiple linear regression was used for analyzing the relationship between EOC with the biomass allocation and root traits. All analyses were conducted using SPSS 14.0 (SPSS Inc., Chicago, USA) and figures were generated by Origin 8.0 (OriginLab Corporation, Northampton, MA, USA).

### Supplementary Information

Supplementary Information.

## Data Availability

All data included in the main text.
