# Supplementary material for: Evaluation of growth adaptation of Cinnamomum camphora seedlings in ionic rare earth tailings environment
Source: Sci Rep. 2023 Oct 7;13:16910. doi: 10.1038/s41598-023-44145-z (PMC10560214; doi:10.1038/s41598-023-44145-z)
Supplement: Supplementary file 1 — Supplementary Information. [file 41598_2023_44145_MOESM1_ESM.pdf]

Plant traits	Total biomass	Stem biomass	Ratio of stem biomass	Leaf biomass	Ratio of leaf biomass	Root biomass	Ratio of root biomass
Stem biomass	0.919**						
Ratio of stem biomass	0.039	-0.116					
Leaf biomass	-0.132	-0.421	0.557				
Ratio of leaf biomass	-0.858	-0.806*	0.008	0.363			
Root biomass	0.929**	0.924**	-0.291	-0.419	-0.838*		
Ratio of root biomass	-0.040	0.143	-0.758*	-0.818*	-0.070	0.322	
Fine root length	0.798*	0.733	0.241	0.037	-0.450	0.698	-0.071
Coarse root length	0.827*	0.822*	-0.213	-0.051	-0.552	0.797*	-0.030
Fine root surface area	0.814*	0.804*	0.113	-0.144	-0.508	0.772*	0.083
Coarse root surface area	0.812*	0.819*	0.029	-0.056	-0.538	0.735	-0.087
Ratio of root surface area	0.187	0.181	0.095	-0.172	-0.200	0.204	0.129
Fine root weight	0.865*	0.868*	-0.329	-0.396	-0.675	0.964**	0.411
Coarse root weight	0.892**	0.887**	-0.288	-0.423	-0.931**	0.941**	0.217
Specific root length of fine root	-0.129	-0.184	0.276	0.172	0.098	-0.204	-0.202
Specific root length of coarse root	-0.527	-0.530	0.125	0.145	0.725	-0.465	0.233
Specific root surface area of fine root	0.203	0.215	0.754	0.182	-0.030	-0.015	-0.420
Specific root surface area of coarse root	-0.211	-0.173	0.057	0.347	0.540	-0.267	-0.177
Specific root surface area of total root	0.039	0.048	0.500	0.218	0.286	-0.080	-0.175

ST1. Correlation coefficients between plant biomass and root trait indexes. Note: **P*<0.05, ***P*<0.01.

Traits	EOC	Oil yield
**Total biomass**	0.206	-0.201
Stem biomass	0.516	0.073
Leaf biomass	0.808*	0.768*
Root biomass	-0.119	-0.450
Fine root length	0.512	0.298
Coarse root length	0.064	-0.204
Fine root surface area	0.355	0.148
Coarse root surface area	0.164	-0.071
Ratio of root surface area	0.212	0.116
Fine root weight	-0.078	-0.316
Coarse root weight	-0.225	-0.619
Ratio of root biomass	0.313	0.404
Specific root length of fine root	0.381	0.344
Specific root length of coarse root	0.190	0.590
Specific root surface area of fine root	0.680	0.635
Specific root surface area of coarse root	0.161	0.408
Specific root surface area of total root	0.636	0.761*

ST2. Correlation analysis of essential oil content with biomass and root morphology indexes in Camphor seedlings. * indicates significant correlation, p<0.05, ** indicates highly significant correlation, P<0.01.
